# Acute health effects of the Tasman Spirit oil spill on residents of Karachi, Pakistan

**DOI:** 10.1186/1471-2458-6-84

**Published:** 2006-04-03

**Authors:** Naveed Zafar Janjua, Pashtoon Murtaza Kasi, Haq Nawaz, Sadia Zohra Farooqui, Urooj Bakht Khuwaja, Najam-ul-Hassan, Syed Nadim Jafri, Shahid Ali Lutfi, Muhammad Masood Kadir, Nalini Sathiakumar

**Affiliations:** 1 Department of Community Health Sciences, the Aga Khan University, Karachi, Pakistan; 2 Sindh Environmental Protection Agency, Karachi, Pakistan; 3 Department of Epidemiology, School of Public Health, University of Alabama at Birmingham, Birmingham, AL, USA

## Abstract

**Background:**

On July 27 2003, a ship carrying crude oil run aground near Karachi and after two weeks released 37,000 tons of its cargo into the sea. Oil on the coastal areas and fumes in air raised health concerns among people. We assessed the immediate health impact of oil spill from the tanker Tasman Spirit on residents of the affected coastline in Karachi, Pakistan.

**Methods:**

We conducted a study consisting of an exposed group including adults living in houses on the affected shoreline and two control groups (A and B) who lived at the distance of 2 km and 20 km away from the sea, respectively. We selected households through systematic sampling and interviewed an adult male and female in each household about symptoms relating to eyes, respiratory tract, skin and nervous system, smoking, allergies, beliefs about the effect on their health and anxiety about the health effects. We used logistic regression procedures to model each symptom as an outcome and the exposure status as an independent variable while adjusting for confounders. We also used linear regression procedure to assess the relationship exposure status with symptoms score; calculated by summation of all symptoms.

**Results:**

Overall 400 subjects were interviewed (exposed, n = 216; group A, n = 83; and group B, n = 101). The exposed group reported a higher occurrence of one or more symptoms compared to either of the control groups (exposed, 96% vs. group A, 70%, group B 85%; *P *< 0.001). Mean summary symptom scores were higher among the exposed group (14.5) than control group A (4.5) and control group B (3.8, *P *< 0.001). Logistic regression models indicated that there were statistically significant, moderate-to-strong associations (Prevalence ORs (POR) ranging from 2.3 to 37.0) between the exposed group and the symptoms. There was a trend of decreasing symptom-specific PORs with increase in distance from the spill site. Multiple linear regression model revealed strong relationship of exposure status with the symptoms score (β = 8.24, 95% CI: 6.37 – 10.12).

**Conclusion:**

Results suggest that the occurrence of increased symptoms among the exposed group is more likely to be due to exposure to the crude oil spill.

## Background

**Figure 1:**
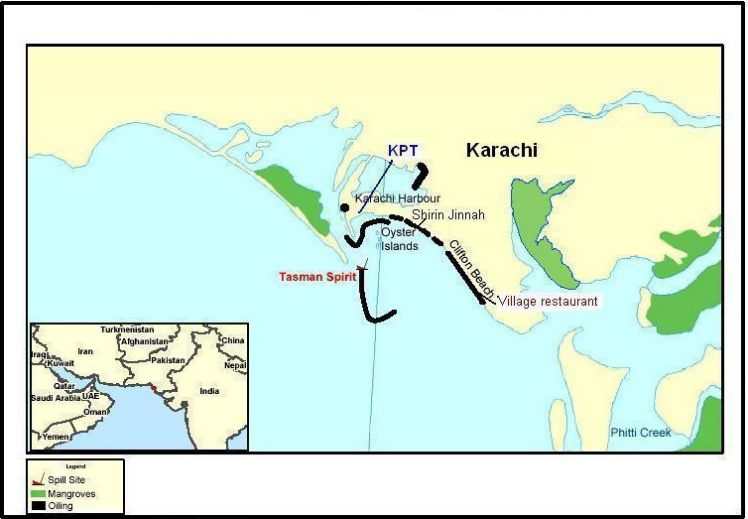
Map of the shoreline covered with crude oil residues after crude oil spill from Tasman Spirit in Karachi, Pakistan 2003 (Source: Sindh Environmental Protection Agency).

On 27 July 2003, the Greek tanker Tasman Spirit, carrying 67000 tons of Iranian light crude oil from Iran to Pakistan, ran aground before entering the harbor channel at the Karachi port, Pakistan. About two weeks later, on the night between 13 and 14 August 2003, the ship broke apart and released its cargo into the sea. Strong winds and rough sea facilitated the spread of oil to about 10 km of residential coastline (Figure 1). After this incident, there were two more episodes of oil spill- the latest being on 29 August 2003, resulting in a total spill of more than 35000 tons of crude oil.

The affected shoreline is a highly populated residential and recreational area. Fumes of volatile organic compounds and mist containing hydrocarbons accompanied by a strong smell dispersed into the residential area. Newspapers and the electronic media provided a wide coverage of the incident and the potential health and environmental impact of the oil spill[1,2]. Newspapers and television channels showed pictures of piles of dead fish and turtles on the oil-covered beach. Extensive media coverage raised concerns about the potential of human health effects among residents in the affected area and among government officials.

Chemical analysis of crude oil from the tanker showed that it contained a high quantity of aromatic hydrocarbons (personal communication Lutfi SA). Crude oil is mixture of many chemicals, the major components being hydrocarbons. Iranian Light Crude oil carried in Tasman Spirit contains high quantity of sulfur (1.35% by wt), 14% light naphtha, 20% heavy naphtha and 4% gasoline and its pour point is -29°C [3]. Aromatic hydrocarbons are probably the main airborne exposure.

Spread of oil and evaporation of volatile contents after spill depends on the physical and chemical properties of the crude oil and the prevailing weather conditions. Low viscosity crude oil spreads rapidly. Low boiling point contents of crude oil evaporate within hours of the spill and if volatile contents are high then evaporation is more. High temperature, high speed wind and strong wave action increase the spread and evaporation of the volatile contents [4]. During August 10^th ^to August 30^th^, 2003 the average maximum temperature in Karachi was 31°C while average minimum temperature was 27°C with the average mean of 29°C [5]. July and August is a rough sea season in Pakistan with strong waves. Moderately high temperature, strong waves and high speed wind [2] moved oil from sea to shoreline and winds carried vapors from sea to land. Initial assessment suggested that about 11,000 tones of volatile organic compounds entered the air after the spill. Assessment of the air revealed that on August 20, VOCs in the polluted air ranged from 44 ppm at Shireen Jinnah Colony to 179 ppm at Village Restaurant.

Residents living in this area were exposed to the high level of volatile organic compounds for at least 15 to 20 days. The level of pollutants gradually tapered off from 8 to 10 ppm of volatile organic compounds on August 30. The pungent smell was perceptible till September 5 at the distance of about 1 km from the sea [6]. These levels suggest high exposure of population to crude oil contents during spill days.

Previous investigations of the Sea Empress, the Nakhodka and the Shetland oil tankers' spills found an increased occurrence of upper respiratory tract irritation, exacerbation of asthma, vertigo, headache, and back and leg pains and psychological ailments among persons living in exposed areas and clean-up workers [7-9].

Studies on acute health effects of exposure to an oil spill are few. This paper is first of such report from a developing country. To address the concerns regarding potential health effects, we undertook an epidemiologic investigation to determine if exposure to the oil spill had resulted in increased acute ill health among the exposed resident population.

## Methods

### Design and study base

The study began on September 1^st ^2003, three weeks after first spill and three days after the last spill. The exact population size of the shoreline is not available; however, it is estimated that about 700,000 people live in this beach town. The beach in this area was covered with thick layer of oil and there were fumes and a mist of oil in the air. The public was prohibited from visiting the beach after spill. However, residents were not asked to evacuate. Exposure was assigned on the basis of geographic location. We defined the exposed group as adults resident in apartments/houses on a 10 km long affected shoreline that extended from Shirin Jinnah colony (near the harbor) to Village restaurant (end of the residential area) and who did not migrate after the oil spill on August 13^th^, 2003. We selected two control groups: control group A living at a distance of 2 km from the affected coastal area and control group B living at a distance of 20 km from the affected shoreline. The distance of 2 km and 20 km were chosen arbitrarily to assess if symptoms depended on the distance from the shoreline. We obtained distance measurements from City District Government of Karachi which provided a list of residential areas at 2 and 20 km, taking the shoreline as a reference point.

### Subject selection

Most of the residential dwellings in both the exposed and control areas were 4 to 10-storeyed buildings with apartments. No official listing of buildings was available through which we could have developed a list of only those buildings that were on the shoreline. There were approximately 100–150 buildings on the shoreline. We selected every 4^th ^building on the shoreline facing towards the beach starting from the Shirin Jinnah colony to the Village restaurant, a total of 25 buildings. From each building we randomly selected one household per floor to assess the effect of elevation on exposure to oil fumes. From each apartment, we selected the first adult male and female we met in the home for interview. Both males and females were included, because of the likelihood of differences in their daily activities and resulting exposure. Typically, men spend more time outdoors and women spend more time indoors. We used the same sampling procedure to select buildings (control group A, 12 building; control group B, 12 buildings), apartment and subjects in control groups. Of 410 persons initially approached, 400 (98%) participated in the study. Of the 400 persons, 216 were in the exposed groups, 83 in control group A and 101 in control group B.

### Interviews and questionnaire

A research team (4 sub-teams comprising of 2 interviewers in each) consisting of physicians and final year medical students conducted face to face interview of participants during household visits. We had an in-office training of the research team on aspects of sampling, consent process, questionnaire and conducting the interviews. We field-tested the questionnaire before actual field work and revised the content as deemed necessary. We developed a questionnaire that included a symptom check list of the known health effects of hydrocarbon exposure based on previous investigations [7,8], adapted from the symptom checklist developed for the Nakhodka oil spill. We specifically asked respondents to report symptoms (as yes/no) that occurred after the first major spill on August 13^th^, 2003. However, we did not ask to specify the exact date of start of a symptom nor when it became better. Major categories of symptoms included ocular (sore, itchy, watery and red eyes), dermal (itchy skin and skin irritation) upper and lower respiratory tract (scratchy throat, sore throat, cough, breathing difficulties, wheeze), gastrointestinal (nausea/vomiting, loss of appetite), central nervous system (headache, dizziness, irritability) and constitutional symptoms (general fatigue, weakness and fever). Besides the detailed symptom list, the questionnaire included information on socio-demographic characteristics, location of house, presence of windows towards sea, active and passive smoking, allergies to chemicals, dust and pollen, course of illness, care sought, and effects on daily life. It also included questions regarding perception about the role of oil spill in producing ill health, perception that if it had affected respondents' health and anxiousness about the effects of oil spill on health [See Additional file 1]. Before the start of interview we obtained a verbal informed consent that included information on the purpose of study, the procedures, confidentiality, risk and benefits and right to withdraw from study any time. We gave a copy of consent from to participants that included phone number and mailing address of principal investigator for additional information and clarification. Study was reviewed by Sindh Environmental Protection Agency and the Department of Community Health Sciences at the Aga Khan University review committee. On returning from the field every day, we assessed the filled questionnaires for completeness. Any vague or inconsistent responses were clarified from the respondents and edited in the questionnaire.

### Statistical analysis

We entered data in MS Access database and analysed it using Statistical Package for Social Sciences (SPSS) version 11. We compared the socio-demographic characteristics of exposed group with the two control groups by the chi-square test for categorical variables and ANOVA for continuous variables. We computed a symptoms score for symptoms relating to oil spill; if a symptom was present we gave a score of "1"otherwise "0" and by summing 48 different symptoms we obtained symptoms score for each subject. We compared symptoms scores among groups using ANOVA. We compared the proportion of symptoms between the exposed and control groups using the prevalence odds ratio (POR) with its corresponding 95% confidence interval (CI). We present results for symptoms with prevalence greater than 30% among the exposed or those have been reported in previous studies to enhance the informativeness of the results. We used logistic regression procedures to adjust for other risk factors. We included a potential risk factor other than crude oil exposure in a logistic regression model if its *P *value was less than 0.20 or if it was biologically meaningful. We assessed the association of exposure status with selected symptoms as outcome variable while adjusting for confounding effect of age, sex, education, history of chemical allergy, dust allergy, active and passive smoking, house on the main road as proxy for exposure to vehicle smoke and anxiety about effect of spill on health. For some variables the numbers in either control group A or B were small, hence we decided not to present adjusted estimates for symptoms with number less than 8 in any of the cell. Goodness of fit of the logistics regression model was assessed using the Hosmer and Lemshow test.

We conducted a subgroup analysis on the exposed group to evaluate the relationship between demographic characteristics and symptoms through logistic regression modeling. The evaluated factors included geographic locations on the basis of residence, age, sex, presence of windows towards sea and house on the main road. Goodness of fit of the logistics regression models was assessed by Hosmer and Lemshow test.

We performed multiple linear regression to assess the relationship between exposure status and the symptoms score while adjusting for age, gender, education, smoking, dust allergy, chemical allergy, and anxiousness about health effect of oil spill. However, only chemical allergy and anxiousness about health effect of oil spill were significant in the final model, hence model included only these two variables beside exposure status. Model assumptions for goodness of fit were assessed using residual analysis that revealed good fit.

## Results

Of the overall 400 participants, 225 (57%) were women and 175 (43%) were men. There were statistically significant differences in the distribution of age, years of education, location of house, self-reported health impact and the number of affected household members between the three groups. The mean age of subjects in control group A (46 years) was higher than the other two groups (exposed group, 35 years; control group B, 31 years) due to a higher proportion of subjects in the > 50 age group and a smaller proportion in the 15–30 age group. Control group B subjects had higher mean years of schooling (12.7 years) than the exposed group (10.3 years) and control group A (11.0 years). A higher proportion of the exposed group (53%) lived in houses on the main road compared to control group A (22%) or B (18%). A higher proportion of the exposed group (78%), a lower proportion of control group A (53%) and a still lower proportion of control group B (24%) reported that they were worried about the possible effects of the oil spill on their health. A higher proportion of exposed group (81%) believed that oil spill had affected their health, while a lower proportion of unexposed group A (23%) and still lower proportion of unexposed group B (3%) believed the same. Differences in smoking habits, passive smoking status, past history of allergy to chemicals, dust and pollen, and past history of wheezing and asthma between the groups were not significantly different (table 1).

Ninety-six percent of the exposed group (207/216), 70% (58/83) of control group A and 85% (86/101) of control group B reported one or more symptoms (*P *< 0.001). Mean symptoms score based on the presence of 48 symptoms in a particular subject were higher among the exposed group (14.1) compared to control A (4.4) or control B (3.8). Further, mean score for each sub-group of symptoms were higher among exposed as compared to control groups. Mean score from nine ocular symptoms among exposed group were 3.4 as compared to1.1 and 0.7 for control groups A and B respectively. Mean score from seven upper respiratory tract symptoms among exposed were 3.1 as compared to 1.1 among each of control group A and B. Mean score from the five skin related symptoms were 1.0 for exposed and 0.1 and 0.1 for each of control group A and B. Mean score from 17 nervous system related symptoms was 4.3 for exposed as compared to 1.2 and 1.3 for control group A and B respectively.

We compared the proportion of each self-reported symptom among the exposed and the control groups, and found that they were highest for the exposed group located closest to the spill, followed by control group A and then B. Proportion of those who reported ever having wheezing was not different among the exposed 8.3% (18/204), the control group A 10.3% (9/84) and the control group B 5% (5/101; *P *= 0.329). Further, exposed 13/204 (6.0 %) who ever had episodes of wheezing that made them short of breath before the spill was not significantly different from control group A 7.2% (6/84) and control group B 3.0% (3/101, *P *= 0.400). After the spill, 6% (13/204) had wheezing with shortness of breath among the exposed while only 1.2% (1/82) among the control A and no one in control B.

Only 32.9 % (68/207) of symptomatic subjects in the exposed group and 29.6% (16/54) of control group A, and 19.0% (16/84) of subjects in control group B consulted a health care provider for the symptoms. Sixty-six percent (45/68) of those who consulted a health care provider in the exposed group reported only one visit. Majority (67%) of the symptomatic subjects in the exposed group reported that they had recovered completely or were recovering, whereas the remaining 33% reported that their illness was persistent.

Logistic regression model adjusted for the effect of age, gender, education, smoking, dust allergy, chemical allergy, house on the main road, and anxiousness about health effect of oil spill indicated that there was statistically significant moderate -to-strong associations (PORs ranging from 2.3 to 37.0) between the exposed group and the symptoms listed above and also reported in table 2. There was a trend of decreasing symptom-specific PORs with decrease in exposure (Table 2).

To assess those who were more affected among the exposed, we constructed logistic regression models for each symptom as dependent variable and included age, sex, building of residence, floor of apartment, house on the main road, and presence of windows towards sea as independent variables. We found that for itchy eyes, scratchy throat, sore throat, nausea/vomiting, headache females were more likely to experience symptoms as compared to males.

Multiple linear regression model revealed strong relationship of exposure with the symptoms score (β = 8.24, 95% CI: 6.37 – 10.12, Table 3).

A higher proportion of those who had symptoms among the exposed group 54.1% (112/207) reported interference of symptoms with their daily routines as compared to 26.8% (15/56) in control group A or 3.5% (3/86) in control group B. The average number of days that the symptomatic subjects were not able to work was significantly higher among exposed group (2.9 ± 6.3) than control A (1.0 ± 3.4) or control B (0.2 ± 1.5, *P *< 0.001).

## Discussion

This study evaluated the acute health effects of a large oil spill on a large urban population. Subjects in the exposed area had a higher occurrence in symptoms than other areas in the vicinity. There was a clear pattern of decreasing symptoms with increasing distance from the incident site. Symptoms involving eyes, throat, skin, headaches and general malaise were some of the commonly reported symptoms.

Knowledge on the health effects of hydrocarbon exposures mainly comes from studies conducted in occupational settings. Contact with petroleum or exposure to its components may result in skin irritation and stinging or redness of eyes, sore throat, breathlessness, nausea, vomiting headache, dizziness or drowsiness [10]. These are the similar symptoms as found in present study. Major hydrocarbons of toxicological interest are benzene, xylene and toulene. Moderately high concentration of these chemicals irritate mucus membranes particularly the eyes [11]. Sulfur containing components of crude oil may cause respiratory difficulty [12].

High environmental level of pollutants and increased occurrence of headache nausea vomiting, eyes symptoms, respiratory difficulties among the exposed as compared to the control groups suggests the role of pollutants in occurrence of these symptoms.

The proportion of those who developed wheezing with shortness of breath was higher among the exposed as compared to the control groups. Previous studies conducted to assess the health effects of oil spill reported an increase in occurrence of sore eyes, sore throat, headache, skin itching and rash, nausea, vomiting and breathing difficulties among those exposed to the vapors of crude oil [7-9,13]. The results of our study are consistent with these results; exposed subjects had increased frequency of sore eyes, sore throat, headache, nausea/vomiting, as compared to the non-exposed group. Breathing difficulties also increased among the exposed group. Before oils pill the proportion of those who ever had wheezing with shortness of breath was not different among the exposed and the control groups. Further, wheezing accompanied with shortness of breath is an acute condition that occurs in bouts after exposure to certain substances which trigger it off. These substances in this particular casewere the crude oil vapors. This further suggest role of exposure in occurrence of asthmatic symptoms.

Self reported disturbance in daily routine due to symptoms further substantiate evidence about the severity of symptoms that was higher among the exposed as compared to the non-exposed groups.

We used distance to assess the degree of exposure by selecting the exposed group form the houses located on the shoreline, control group A at 2 km from the source of exposure and control group B at about 20 km from the source. The frequency of exposure as well as strength of associations for many symptoms decreased with the increase in distance. The presence of dose response also suggests a relationship between occurrence of symptoms and exposure.

Those who were exposed for a longer period of time like women among the exposed group, were more likely to develop nausea, vomiting, headache, itchy eyes, scratchy throat and sore throat as compared to men. Women spent most of their time at homes as most of them are housewives, while men were away during the day at their jobs, resulting in an increased exposure to women. These symptoms occurred after exposure to hydrocarbons [10,11]. In other studies those who were exposed for longer period of time experienced more symptoms as compared to those who were exposed for lesser duration [7].

We analyzed data using two different approaches- multiple linear regression using symptoms score as dependent variable and logistic regression using individual symptom as dependent variable. Both of these approaches revealed strong association of exposure with the symptoms. This provides additional strength to evidence of relationship between exposure to oil spill and ill health.

### Strengths and limitations

Strengths include the first study on oil spill health effects to be reported from a developing country, the high participation rate, and collection information on confounders and use of analytical procedure to control the effect of confounders.

Oil spills and other environmental disasters have been reported to increase anxiety, distress and depression among the exposed [14,15]. However this also depends on level of coverage by the media, concern among the population and availability of means that create awareness and concern among the population. The population affected in current spill has higher educational level than rest of Pakistan but strong civil society organizations that create concerns/awareness are not present. Therefore, level of anxiety may be lesser than that have beenreported from developed countries. We did not have a validated questionnaire to capture anxiety. However, we accounted for anxiety using a single question on anxiousness about effect of oil spill on health.

Since information was based on questionnaires administered at one point in time, there is a likelihood of symptoms being present before the spill occurred. This is the problem due to nature of design used in such studies. However, we addressed this possibility by attempting to determine the occurrence of symptoms related to the specific date of incident. The short recall and correlation of symptoms with a specific exposure facilitated the accuracy of the information. Further, there was the potential for recall bias and over-reporting of symptoms by the exposed group because exposed group may become anxious about their health and work. We addressed the potential problem of differential recall by eliciting information on anxiousness about health due to oil spill and adjusting for this in analysis.

Sample size was based on the availability of funds for conducting this investigation rather than statistical considerations. This has resulted in smaller number of participants in the study that is reflected in our results with small numbers for some variables and resulting wide confidence intervals.

We used three different geographic locations to represent distance form the source of exposure to assign exposure status; this is a crude exposure measurement. Environmental levels of pollutants or biomonitoring could have provided better exposure assessment. However, since exposure was only from one source; distance from the source represents adequate proxy for exposure.

## Conclusion

This study shows a possible relationship between the oil spill and acute symptoms among residents near the spill, indicating an adverse effect on their health. Efforts should be devoted to prevent future occurrence of such incidents through instituting an emergency response and preparedness system. Long term health impact of the crude oil spill exposure should be investigated.

## Competing interests

The author(s) declare that they have no competing interests.

## Authors' contributions

NZJ conceived the idea, designed the study, did analysis and prepared the manuscript. PMK, HN, SZF, UBK, NH, SNJ, SL and MMK contributed in design and conduct of study. PMK, MMK, NS contributed in manuscript preparation and revisions. All authors read and approved the final version of manuscript.

## Pre-publication history

The pre-publication history for this paper can be accessed here:



## Supplementary Material

Additional File 1 The file contain questionnaire in MS word format used in the study to collect information from the study participants. Click here for file

## Figures and Tables

**Table 1:** Selected characteristics, medical history and beliefs of study participants according to exposure category for health effect of oil spill in Karachi, Pakistan 2003

**Variables**	**Exposed group**		**Control group A**		**Control group B**		
	**n**	**%**	**n**	**%**	**n**	**%**	***P***^a^

Total	216	100	83	100	101	100	
							
Gender							
Female	123	57	50	60	52	52	0.47
Male	93	43	33	40	49	48	
							
Age group (years)							
15–30	95	44	20	24	64	63	<0.001
31–50	87	40	26	31	27	27	
>50	34	16	37	45	10	10	
Mean (SD)	35.5 (14.3)		45.5 (16.4)		31.2 (13.3)		<0.001
							
Education (years)							
0	38	18	10	12	6	6	0.05
1–10	47	22	18	22	13	13	
11–12	44	20	17	21	23	23	
13–14	48	22	19	23	32	32	
>14	39	18	19	23	27	27	
Mean (SD)	10.3 (5.5)		12.7 (3.8)		11.0 (5.1)		<0.001
							
Employment status							
Housewife	88	40.7	35	42.2	13	12.9	<0.001
Student	28	13.0	8	9.6	26	25.7	
Employed	100	46.3	40	48.2	62	61.4	
							
House on main road							
Yes	115	53	18	22	18	18	<0.001
No	101	47	65	78	83	82	
							
Smoking							
Yes	36	17	12	14	14	14	0.78
No	180	83	71	86	87	86	
							
Passive smoking							
Yes	46	21	15	18	17	17	0.60
No	170	79	68	82	84	83	
							
Allergy to chemicals							
Yes	59	27	22	26	17	17	0.12
No	157	73	61	74	84	83	
							
Allergy to dust							
Yes	78	36	32	39	26	26	0.12
No	138	64	51	61	75	74	
							
Allergy to pollen							
Yes	16	7	6	7	0	0	_
No	200	93	77	93	101	100	
							
Ever had wheezing							
Yes	18	8	9	11	5	5	0.33
No	198	92	74	89	96	95	
							
Ever had wheezing with shortness of breath							
Yes	13	6	6	7	3	3	0.40
No	203	94	77	93	98	97	
							
Belief that oil fumes can cause illness							
Yes	195	90	69	83	94	93	0.28
No	15	7	10	12	5	5	
Don't know	6	3	4	5	2	2	
							
Worried about effect of oil spill on health							
Yes	168	78	44	53	24	24	<0.001
No	48	22	39	47	77	76	
							
Fumes have affected health							
Yes	175	81	19	23	3	3	<0.001
No	41	19	64	77	98	97	
							
Mean number of ill household members (SD)	2.5 (2.2)		0.9 (1.9)		0.3 (0.8)		<0.001

^a^*P*, chi-square test comparing proportion between the groups or ANOVA (F test) comparing the means between the groups.

**Table 2:** Crude and adjusted ^a ^prevalence odds ratio (POR) and corresponding 95% confidence interval (CI) for specific symptoms by exposure category group

**Symptoms **	**N**	**Pr ^b^**	**Crude POR**	**95% CI**	**Adjusted POR**	**95% CI**
**Ocular Symptoms**						
Sore eyes						
Control B	12	11.9	1.0	---	1.0	---
Control A	20	24.1	2.4	1.1–5.2	2.4	1.0–5.6
Exposed	132	61.1	11.7	6.0–22.6	11.9	5.7–24.9
						
Itchy eyes						
Control B	16	15.8	1.0	---	1.0	---
Control A	18	21.7	1.5	0.7–3.1	1.5	0.7–3.4
Exposed	118	54.6	6.4	3.5–11.6	5.9	3.0–11.7
						
Watery eyes						
Control B	13	12.9	1.0	---	1.0	---
Control A	16	19.3	1.6	0.7–3.6	1.5	0.6–3.5
Exposed	103	47.7	6.2	3.2–11.7	5.3	2.6–10.9
						
Red eyes						
Control B	8	7.9	1.0	---	1	---
Control A	12	14.5	2.0	0.8–5.1	2.4	0.9–6.6
Exposed	90	41.7	8.3	3.8–18.0	8.5	3.6–19.7
**Respiratory symptoms**						
Dry throat						
Control B	25	24.8	1.0	---		---
Control A	22	26.5	1.1	0.6–2.1	0.9	0.5–1.9
Exposed	140	64.8	5.6	3.3–9.5	4.7	2.5–8.6
						
Scratchy throat						
Control B	22	21.8	1.0	---	1.0	---
Control A	18	21.7	1.0	0.5–2.0	0.8	0.4–1.7
Exposed	129	59.7	5.3	3.1–9.2	3.5	1.9–6.5
						
Sore throat						
Control B	16	15.8	1.0	---	1.0	---
Control A	18	21.7	1.5	0.7–3.1	1.2	0.5–2.7
Exposed	113	52.3	5.8	3.2–10.6	3.8	1.9–7.4
						
Cough						
Control B	18	17.8	1.0	---	1.0	---
Control A	12	14.5	0.8	0.4–1.7	0.6	0.2–1.4
Exposed	94	43.5	3.6	2.0–6.3	2.3	1.2–4.4
Shortness of breath after spill ^c^						
Control B	2	1.0	---			---
Control A	15	18.1	10.9	2.4–49.3		
Exposed	110	50.9	51.4	12.4–213.6		
						
Difficulty in breathing after spill						
Control B	0	0.0	---		---	
Control A	1	1.2				
Exposed	22	10.2				
						
Wheezing after spill						
Control B	0	0	---		---	
Control A	8	9.6				
Exposed	42	19.4				
						
Wheezing with shortness of breath after spill						
Control B	0	0	---		---	
Control A	1	1.2				
Exposed	13	6.0				
						
**Skin symptoms**						
Itchy Skin ^c^						
Control B	5	5.0	1.0	---		---
Control A	6	7.2	1.5	0.4–5.1		
Exposed	75	34.7	10.2	4.0–26.1		
						
Skin irritation ^c^						
Control B	4	4.0	1.0	---		---
Control A	2	2.4	0.6	0.1–3.4		
Exposed	54	25.0	8.1	2.8–23.0		
						
**Nervous system symptoms**						
Nausea/vomiting^c^						
Control B	7	6.9	1.0	---		---
Control A	12	14.5	2.3	0.9–6.1		
Exposed	84	38.9	8.5	3.8–19.3		
						
Headache						
Control B	29	28.7	1.0	---	1.0	---
Control A	18	21.7	0.7	0.3–1.4	0.6	0.3–1.2
Exposed	138	63.9	4.4	2.6–7.3	3.1	1.7–5.5
						
Dizziness ^c^						
Control B	7	6.9	1.0	---		---
Control A	9	10.8	1.6	0.6–4.6		
Exposed	75	34.7	7.1	3.2–16.2		
						
Irritability						
Control B	4	4.0	1.0	---	1.0	---
Control A	7	8.4	2.2	0.6–7.9	1.6	0.4–5.9
Exposed	77	35.6	13.4	4.8–37.9	11.3	3.8–34.2
						
Weakness of extremities ^c^						
Control B	4	4.0	1.0	---		---
Control A	12	14.5	0.5	0.2–1.3		
Exposed	91	42.1	2.6	1.4–4.9		
						
**Other symptoms**						
Fever						
Control B	9	8.9	1.0	---	1.0	---
Control A	7	8.4	0.9	0.3–2.6	1.0	0.4–3.1
Exposed	65	30.1	4.4	2.1–9.3	4.5	1.9–10.3
						
Loss of appetite ^c^						
Control B	9	8.9	1.0	---		---
Control A	4	4.8	0.5	0.2–1.7		
Exposed	72	33.3	5.1	2.4–0.7		
						
General fatigue						
Control B	27	26.7	1.0	---	1.0	---
Control A	22	26.5	1.0	0.5–1.9	0.9	0.4–1.8
Exposed	116	53.7	3.2	1.9–5.3	3.1	1.7–5.7

^a ^Models adjusted for age, gender, education, smoking, dust allergy, chemical allergy, house on the main road and anxiousness about health effect of oil spill.

^b ^Pr = number of subjects with the specific symptom in the specific exposure category/total number of subjects in that category.

^c ^Numbers in some cells are small, hence adjusted estimates may not be reliable.

**Table 3:** Multiple linear regression model of relationship between exposure status and the symptoms score

**Variables**	**β**	**95% CI for β**
		
Exposed	8.24	6.37 – 10.12
Control A	-0.66	-2.76 – 1.45
Anxiousness about health effects of oil spill	3.19	1.61 – 4.78
Chemical Allergy	3.79	2.17 – 5.41
Intercept	2.36	0.91 – 3.80
