# Supplementary material for: Acute health effects of the Tasman Spirit oil spill on residents of Karachi, Pakistan
Source: BMC Public Health. 2006 Apr 3;6:84. doi: 10.1186/1471-2458-6-84 (PMC1484477; doi:10.1186/1471-2458-6-84)
Supplement: Additional File 1 — The file contain questionnaire in MS word format used in the study to collect information from the study participants. [file 1471-2458-6-84-S1.doc]

# Oil Spill Health Effect Questionnaire for Residents

**I D___ ___ ___ ___** Area: 1. Sea area 2. Control **A**  3. Control **B**

**Informed Consent: 0 Interviewed by: ________________**

**Signature: ____________________ Date: ____/____/______**

**Socio-demographic characteristics**

1. Name ______________ 2. Age (yrs): ________3. Gender: 1. Male 2. Female

4. Address ___________________________________________ 5. Tel: _____________

5. Education (completed years) ___________ 6. Occupation__________________

6. Monthly family income (Rs.) _____________

*Now I would like to ask some questions about your health*

| # | Question | Code | Answer |
| --- | --- | --- | --- |
| 7 | Have you ever had asthma? Whistling/wheezing *(if no skip Q8-9)* | 1. Yes 2. No |  |
| 8 | Have you received medication by doctor for this | 1. Yes 2. No |  |
| 9 | Have you ever had an ATTACK of wheezing that has made you feel short of breath? | 1. Yes 2. No |  |
| 10 | Did you have any attack of 1. difficulty in breathing 2. wheezing /whistling 3. SOB  after oil spill? | 1. Difficulty in breathing 2. Wheezing /whistling 3. SOB  4. No |  |
| 11 | Have you required medicine or treatment for the(se) attack(s)? | 1. Yes 2. No |  |

| **Q.12: Did you have any of the following symptoms after oil spill on August 13?** | | | | | |
| --- | --- | --- | --- | --- | --- |
| *(Multiple symptoms can occur mark all that happened)* | | | | | |
| 1. Sore eyes | 1.Yes 2. No |  | 22. Dizziness | 1.Yes 2. No |  |
| 2. Jiggling of vision | 1.Yes 2. No |  | 23. Nausea and vomiting | 1.Yes 2. No |  |
| 3. Itchy eyes | 1.Yes 2. No |  | 24. Dull headache, headache | 1.Yes 2. No |  |
| 4. Eyestrain | 1.Yes 2. No |  | 25. Palpitation | 1.Yes 2. No |  |
| 5. Injection of conjunctiva | 1.Yes 2. No |  | 26. Loss of appetite | 1.Yes 2. No |  |
| 6. Puffy eyes | 1.Yes 2. No |  | 27. Stomachache | 1.Yes 2. No |  |
| 7. Teary eyes | 1.Yes 2. No |  | 28. Abdominal pain | 1.Yes 2. No |  |
| 8. Eye mucus | 1.Yes 2. No |  | 29. Diarrhea | 1.Yes 2. No |  |
| 9. Poor vision | 1.Yes 2. No |  | 30. Constipation | 1.Yes 2. No |  |
|  |  |  | 31. General Fatigue | 1.Yes 2. No |  |
| 10. Runny nose | 1.Yes 2. No |  | 32. Fever | 1.Yes 2. No |  |
| 11. Itchy nose | 1.Yes 2. No |  |  |  |  |
|  |  |  | 33. Tingling of extremities | 1.Yes 2. No |  |
| 12. Dry throat | 1.Yes 2. No |  | 34. Tremor of extremities | 1.Yes 2. No |  |
| 13. Scratchy throat | 1.Yes 2. No |  | 35. Weakness of extremities | 1.Yes 2. No |  |
| 14. Sore throat | 1.Yes 2. No |  | 36. Insomnia | 1.Yes 2. No |  |
| 15. Phlegm | 1.Yes 2. No |  | 38 Loss of concentration | 1.Yes 2. No |  |
| 16. Cough | 1.Yes 2. No |  |  |  |  |
|  |  |  | 39. Irritability | 1.Yes 2. No |  |
| 17. Skin swelling | 1.Yes 2. No |  | 40. Leg pain or low back pain | 1.Yes 2. No |  |
| 18. Itchy skin | 1.Yes 2. No |  | 41. Injury | 1.Yes 2. No |  |
| 19. Eruption or blister | 1.Yes 2. No |  |  |  |  |
| 20. Flushed face | 1.Yes 2. No |  |  |  |  |
| 21. Skin irritation | 1.Yes 2. No |  |  |  |  |

| 13 | How you can best explain the course of the illness after oil spill  *(For only those who reported any symptom after spill)* | 1. Same as before  2. Illness replaced after getting better  3. Getting better with time  4. Getting worse with time  00. Any other |  |
| --- | --- | --- | --- |
| 14 | Did you seek care for these complaints? | 1. Yes 2. No |  |
| 15 | How many time you visited physician after oil spill? |  |  |
| 16 | How much you paid for the seeking care and medications? *(Include fee and medicine)* |  |  |
| 17 | Did these symptoms interfere with your daily routine? *(Like absence from work, school)* | 1. Yes 2. No |  |
| 18 | How many days you were not able to work? |  |  |
| 19 | Which symptom was most distressing of all? |  |  |
| 20 | Has fumes and smell from oil spill affected your daily routine | 1. No 2. Yes, explain_______ |  |
| 21 | How far is house from the sea? (in meters) |  |  |
| 22 | Did you have windows that open towards sea? |  |  |
| 23 | Which direction does the house open? | 1. West  2. East  3. Others |  |
| 24 | Is house on the main road? | 1. Yes 2. No |  |
| 25 | Distance in meter between the main road and house(*main road is having cross-section, where minibuses passes)* |  |  |
| 26 | Is this road heavily traveled?  *Vehicle passes…* | 1. Every minute  2. Every 5 minutes  3. Every 10 minutes  4. More than 10 minutes |  |
| 27 | Do you smoke? | 1. Yes 2. No |  |
| 28 | For how many years? |  |  |
| 29 | How many cigarettes per day? |  |  |
| 30 | Any one else in the house smoke? | 1. Yes 2. No |  |
| 31 | How many cigarettes per day? |  |  |
|  | **History of allergies** |  |  |
| 32 | Are you allergic/ develop wheezing upon exposure to pungent chemicals? | 1. Yes 2. No 3. DK |  |
| 33 | Are you allergic/ develop wheezing upon exposure to dust? | 1. Yes 2. No 3. DK |  |
| 34 | Are you allergic/ develop wheezing upon exposure to pollens | 1. Yes 2. No 3. DK |  |
| 35 | In your home did any one have allergies? | 1. Yes 2. No 3. DK |  |
|  | **Health perception after oil spill** |  |  |
| 36 | Do you think fumes from oil spill can produce any illness? | 1. Yes 2. No |  |
| 37 | Do you think oil spill has caused you ill? | 1. Yes 2. No |  |
| 38 | Are you anxious/worried about the ill health effect of the oil spill? | 1. Yes 2. No |  |
| 39 | How many people fell ill in your home? |  |  |

Thank you very much for yout time and cooperation
